# Prognostic utility of differential tissue characterization of cardiac neoplasm and thrombus via late gadolinium enhancement cardiovascular magnetic resonance among patients with advanced systemic cancer

**DOI:** 10.1186/s12968-017-0390-2

**Published:** 2017-10-12

**Authors:** Angel T. Chan, Andrew J. Plodkowski, Shawn C. Pun, Yuliya Lakhman, Darragh F. Halpenny, Jiwon Kim, Samantha R. Goldburg, Mathew J. Matasar, Chaya S. Moskowitz, Dipti Gupta, Richard Steingart, Jonathan W. Weinsaft

**Affiliations:** 1 Departments of Medicine, Memorial Sloan Kettering Cancer Center, New York, NY USA; 2 Radiology, Memorial Sloan Kettering Cancer Center, New York, NY USA; 3 Epidemiology and Biostatistics, Memorial Sloan Kettering Cancer Center, New York, NY USA; 4 Department of Medicine, Weill Cornell Medical College, 525 East 68th Street, New York, NY 10021 USA

**Keywords:** Cardio-oncology, Cardiac metastases, Cardiac thrombus, Cardiac mass

## Abstract

**Background:**

Late gadolinium enhancement (LGE-) cardiovascular magnetic resonance (CMR) is well-validated for cardiac mass (C_MASS_) tissue characterization to differentiate neoplasm (C_NEO_) from thrombus (C_THR_): Prognostic implications of C_MASS_ subtypes among systemic cancer patients are unknown.

**Methods:**

C_MASS_ + patients and controls (C_MASS_ -) matched for cancer diagnosis and stage underwent a standardized CMR protocol, including LGE-CMR (IR-GRE) for tissue characterization and balanced steady state free precession cine-CMR (SSFP) for cardiac structure/function. C_MASS_ subtypes (C_NEO_, C_THR_) were respectively defined by presence or absence of enhancement on LGE-CMR; lesions were quantified for tissue properties (contrast-to-noise ratio (CNR); signal-to-noise ratio (SNR) and size. Clinical follow-up was performed to evaluate prognosis in relation to C_MASS_ etiology.

**Results:**

The study population comprised 126 patients with systemic neoplasms referred for CMR, of whom 50% (*n* = 63) had C_MASS_ + (C_NEO_ = 32%, C_THR_ = 18%). Cancer etiology differed between C_NEO_ (sarcoma = 20%, lung = 18%) and C_THR_ (lymphoma = 30%, GI = 26%); cardiac function (left ventricular ejection fraction: 63 ± 9 vs. 62 ± 10%; *p* = 0.51∣ right ventricular ejection fraction: 53 ± 9 vs. 54 ± 8%; *p* = 0.47) and geometric indices were similar (all p = NS). LGE-CMR tissue properties assessed by CNR (13.1 ± 13.0 vs. 1.6 ± 1.0; *p* < 0.001) and SNR (29.7 ± 20.4 vs. 15.0 ± 11.4, *p* = 0.003) were higher for C_NEO_, consistent with visually-assigned diagnostic categories. C_THR_ were more likely to localize to the right atrium (78% vs. 25%, p < 0.001); nearly all (17/18) were associated with central catheters. Lesion size (17.3 ± 23.8 vs. 2.0 ± 1.5 cm^2^; p < 0.001) was greater with C_NEO_ vs. C_THR_, as was systemic disease burden (cancer-involved organs: 3.6 ± 2.0 vs. 2.3 ± 2.1; *p* = 0.02). Mortality during a median follow-up of 2.5 years was markedly higher among patients with C_NEO_ compared to those with C_THR_ (HR = 3.13 [CI 1.54–6.39], *p* = 0.002); prognosis was similar when patients were stratified by lesion size assessed via area (HR = 0.99 per cm^2^ [CI 0.98–1.01], *p* = 0.40) or maximal diameter (HR = 0.98 per cm [CI 0.91–1.06], *p* = 0.61). C_THR_ conferred similar mortality risk compared to cancer-matched controls without cardiac involvement (*p* = 0.64) whereas mortality associated with C_NEO_ was slightly higher albeit non-significant (*p* = 0.12).

**Conclusions:**

Among a broad cancer cohort with cardiac masses, C_NEO_ defined by LGE-CMR tissue characterization conferred markedly poorer prognosis than C_THR_, whereas anatomic assessment via cine-CMR did not stratify mortality risk. Both C_NEO_ and C_THR_ are associated with similar prognosis compared to C_MASS_ - controls matched for cancer type and disease extent.

## Background

Patients with systemic cancer are at substantial risk for development of cardiac masses (C_MASS_), including cardiac neoplasm (C_NEO_) and thrombus (C_THR_) [1–5]. Differentiation between C_NEO_ and C_THR_ impacts therapeutic decision-making, including use of anti-cancer therapies and anticoagulation. However, discrimination between the two based on anatomic appearance alone can be challenging, as C_NEO_ and C_THR_ can be similar in size and shape. Given the need to target therapeutic approaches and stratify prognosis in relation to C_MASS_ etiology, accurate differentiation between C_NEO_ and C_THR_ is of substantial importance.

One approach to discriminate between neoplasm and thrombus stems from tissue properties relating to presence or absence of vascular supply. C_NEO_ requires vascularity for tumorigenesis, whereas C_THR_ can be intrinsically defined based on avascularity. Late gadolinium enhancement cardiovascular magnetic resonance (LGE-CMR) imaging enables C_NEO_ to be differentiated from C_THR_ based on vascular composition. Prior research by our group and others has validated LGE-CMR as a highly accurate test for thrombus among non-cancer cohorts, including post-myocardial infarction and heart failure patients in whom LGE-CMR evidenced left ventricular (LV) thrombus has been shown to correlate with histopathology findings, and yield incremental utility (compared to anatomic imaging) for stratification of thrombo-embolic events [6–9]. More recently, we have employed LGE-CMR tissue characterization to identify C_NEO_ among patients with advanced systemic cancer, among whom prognosis paralleled cancer etiology and systemic disease burden [3]. However, prior research to date has been limited to patient cohorts with *either* C_NEO_ *or* C_THR_, thereby prohibiting comparison of risk factors and differential prognosis associated with each of these two conditions.

This study employed LGE-CMR tissue characterization to assess C_NEO_ and C_THR_ among a broad cohort of at-risk patients with systemic cancer. Study aims were as follows: (1) identify cancer-associated risk factors predisposing to C_NEO_ and C_THR_; (2) compare anatomic location, function sequelae, and contrast-enhanced tissue properties of C_NEO_ and C_THR_; and (3) assess relative prognostic implications of C_NEO_ and C_THR_ compared to controls matched for cancer etiology and extra-cardiac disease burden.

## Methods

### Study population

The population included adults (≥18 years old) with systemic neoplasms with and without evidence of C_MASS_ as identified by late gadolinium enhancement (LGE-) CMR: C_MASS_ was defined as a discrete tissue prominence within either a cardiac chamber or pericardium, which demonstrated distinct enhancement pattern from surrounding myocardium. Patients with liquid tumors (i.e. leukemia) as well as primary cardiac malignancies were excluded. Established criteria [3, 7–9] were used to distinguish C_MASS_ subtypes: (1) Neoplasm (C_NEO_) was defined as C_MASS_ with evidence of vascularity on LGE-CMR, defined by heterogeneous or diffuse contrast enhancement. (2) Thrombus (C_THR_) was defined as C_MASS_ without contrast enhancement. C_MASS_ + patients (i.e. C_NEO_ and C_THR_) were each matched (1:1) with patients with no cardiac mass (C_MASS_ -) on LGE-CMR but equivalent primary cancer etiology and disease stage.

**Fig. 1:**
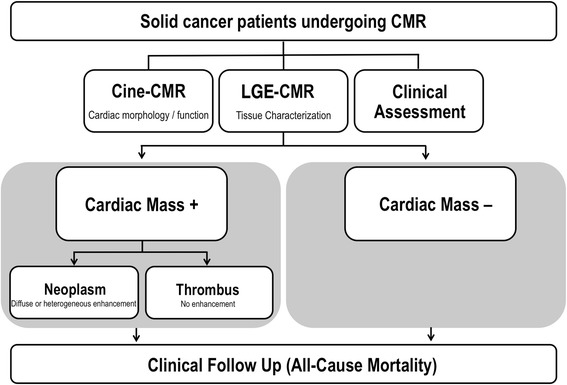
Study Design. Schematic of overall study design, inclusive of baseline LGE-CMR (for mass tissue characterization) and subsequent clinical follow-up (for all cause mortality). Note that for all C_MASS_ + patients, etiology (C_NEO_ vs. C_THR_) was established based on presence or absence of enhancement on LGE-CMR

Figure 1 provides an overall schematic of the research protocol. In all patients, comprehensive clinical data were collected in a standardized manner, including cancer etiology, coronary heart disease risk factors, and anti-cancer therapies administered within 6 months of CMR. C_MASS_ data (imaging and clinical assessment) was collected as part of an ongoing registry of patients undergoing clinically indicated CMR, for which initial results (limited to C_NEO_ patients) have been partially reported [3]. CMR was performed between September 2012 and January 2017 at Memorial Sloan Kettering Cancer Center (New York, New York, USA). Mortality status after CMR was assessed via review of electronic medical records so as to test prognosis in relation to presence and pattern of C_MASS_.

This study entailed analysis of imaging and ancillary data acquired for primarily clinical purposes; no dedicated interventions (imaging or otherwise) were performed for exclusively research purposes. Ethics approval for this protocol was provided by the Memorial Sloan Kettering Cancer Center Institutional Review Board, which approved a waiver of informed consent for analysis of pre-existing clinical data.

### CMR protocol

CMR was performed on commercial (1.5 T [89%], 3.0 T [11%]) scanners (General Electric Healthcare, Waukesha, Wisconsin, USA). Exams included cine- and LGE-CMR, both of which were obtained in contiguous LV short-axis (from mitral annulus through the apex) and long-axis (2, 3, 4 chamber) imaging orientations. Cine-CMR utilized a balanced steady-state free precession (bSSFP) pulse sequence. LGE-CMR utilized an inversion recovery pulse sequence; images were acquired following gadolinium (0.2 mmol/kg) infusion. Conventional (inversion time [TI] ~300 msec) and “long TI” (TI 600 msec) were used to discern C_MASS_ vascularity concordant with prior methods applied and validated by our group [3]: Conventional TI LGE-CMR was acquired uniformly in all patients; additional breath holds required for supplemental long TI LGE-CMR were tolerated in 97% (61/63) of C_MASS_ + patients (100% C_THR_, 95% C_NEO_).

### Image analysis

#### C_MASS_

**Fig. 2:**
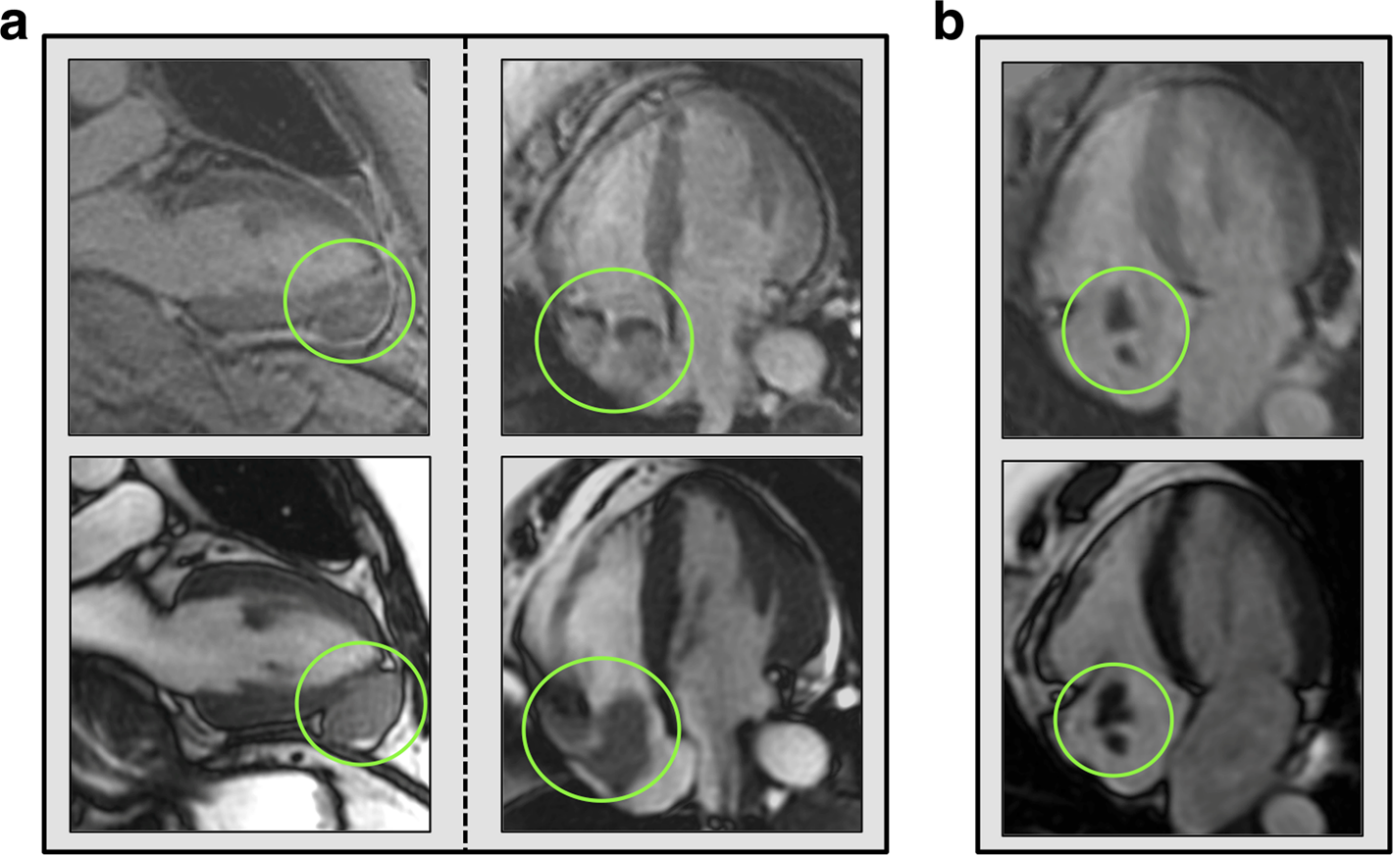
C_MASS_ Enhancement Patterns Identified by LGE-CMR. **a** C_NEO_: Representative examples of diffuse (left) and heterogeneous (right) enhancement as manifest on (long TI) LGE-CMR (lesions denoted within green circles). Corresponding cine-CMR images shown on bottom for purpose of anatomic localization. Both lesions (diffusely enhancing pericardial lesion adjacent to distal left ventricle (LV), heterogeneously enhancing right atrial (RA) lesion) identified in patients with advanced (stage IV) melanoma. **b** C_THR_: Typical non-enhancing lesion deemed consistent with avascular composition (thrombus). Note that RA localization of lesion, which was identified by LGE-CMR following placement of central catheter for therapeutic management of stage IV ovarian cancer

Whereas C_THR_ was intrinsically defined based on uniform absence of contrast uptake, C_NEO_ lesions were categorized based on two distinct enhancement patterns: Heterogeneous lesions manifested both discrete hyper- and hypoenhancement within a single mass; diffuse lesions manifested diffuse enhancement throughout the entire mass. Figure 2 provides representative examples of C_MASS_ enhancement patterns on LGE-CMR.

Quantitatively signal-to-noise (SNR) and contrast-to-noise (CNR) ratios on (long-TI) LGE-CMR were also used to assess enhancement patterns. Analyses were performed concordant with established methods previously applied by our group [3]. For patients with multiple lesions, the largest mass (based on cumulative LGE-CMR review) was used for quantitative image analysis.

C_NEO_ and C_THR_ were scored in a binary manner (present or absent), and localized based on chamber location (right atrium [RA], right ventricle [RV], left atrium [LA], LV) or pericardial involvement. Anatomic and functional properties of lesions were measured on cine-CMR, including lesion size (area, perimeter, and orthogonal linear dimensions), border irregularity (perimeter/shortest orthogonal diameter), valvular adherence/regurgitation, and ventricular outflow tract obstruction.

#### Cardiac chamber geometry

Cine-CMR was used to measure cardiac structure and function, as well as to identify pericardial and pleural effusions. LV and RV chamber volumes and ejection fraction (EF) were quantified based on planimetry of end-diastolic and end-systolic short axis slices. LV mass (including papillary muscles and trabeculae) was measured at end-diastole. LA and RA areas were measured during atrial end-diastole in 4-chamber orientation.

### Mode of spread and prognostic assessment

Clinical documentation and extra-cardiac imaging (within 6 months of CMR) were reviewed to evaluate overall tumor burden. Extent of metastatic disease (outside of primary cancer organ) was evaluated in accordance with established methods based on number of major organ systems involved (central nervous system, head/neck, lung, pleura, liver, gastrointestinal, genitourinary, bones/soft tissue, thoracic and abdominal lymph nodes); a cumulative scoring system was used with each organ system assigned one point [10–12]. Electronic medical records were reviewed to assess all-cause mortality status. Time to event (death) was calculated in relation to CMR.

### Statistical methods

Comparisons between groups with or without C_MASS_, as well as between C_MASS_ subtypes (C_NEO_ vs C_THR_) were made using Student’s t-test (expressed as mean ± standard deviation) for continuous variables, and Chi-square or Fishers exact tests for categorical variables: Paired testing (e.g. paired t-test or McNemar’s test) were employed for matched case-control comparisons. The Kaplan-Meier method estimated the survival function. Cox proportional hazards model with a shared gamma frailty were used to compare mortality risk between groups adjusting for the matching. Receiver operating characteristics (ROC) analysis was used to evaluate overall diagnostic test performance of given imaging parameters (e.g. lesion size, SNR, CNR) for differentiation between LGE-CMR designated C_NEO_ and C_THR_, and to derive cutoffs for maximal sensitivity and specificity. Statistical calculations were performed using SPSS 24.0 (SPSS Inc. [International Business Machines, Inc., Armonk, New York, USA]) and Stata 13.0 for Windows. Two-sided *p* < 0.05 was considered indicative of statistical significance.

## Results

### Population characteristics

The study population comprised 126 patients with systemic neoplasms undergoing CMR, including 63 with cardiac masses (C_MASS_). Table 1 reports clinical and imaging characteristics of the population, including comparisons between C_MASS_ affected patients and matched controls, as well as between affected patients within each C_MASS_ subtype (C_NEO_, C_THR_). As shown, C_MASS_ + patients had a slightly higher burden of extra-cardiac disease as assessed based on number of cancer-affected organ systems (*p* = 0.02), but were similar with respect to age, gender, as well as cardiac remodeling and functional indices (all *p* = NS). Cancer subtype was verified by pathology in all patients; 13% (*n* = 5) of patients with C_NEO_ underwent tissue-based verification of mass etiology: Results demonstrated uniform concordance between biopsy and CMR-designation of C_NEO_ based on mass-associated contrast-enhancement.

**Table 1:** Population Characteristics

	Overall (*n* = 126)	C_MASS_ + (n = 63)	C_MASS_ - (n = 63)	p	C_MASS_ +		p
					C_NEO_ (*n* = 40)	C_THR_ (*n* = 23)	
Clinical Characteristics							
Age (years)	57 ± 15	57 ± 15	56 ± 16	0.58	60 ± 14	53 ± 16	0.10
Male gender	56% (70)	54% (34)	57% (36)	0.85	55% (22)	52% (12)	0.83
Body Surface Area (m^2^)	1.8 ± 0.3	1.8 ± 0.3	1.9 ± 0.3	0.49	1.8 ± 0.3	1.8 ± 0.2	0.66
Leading Cancer Etiologies^a^							
Gastrointestinal	19% (24)	19% (12)	19% (12)	1.00	15% (6)	26% (6)	0.33
Sarcoma	16% (20)	16% (10)	16% (10)	1.00	20% (8)	9% (2)	0.30
Lymphoma	14% (18)	14% (9)	14% (9)	1.00	5% (2)	30% (7)	0.009
Lung	14% (18)	14% (9)	14% (9)	1.00	18% (7)	9% (2)	0.47
Genitourinary	13% (16)	13% (8)	13% (8)	1.00	13% (5)	13% (3)	1.00
Cancer Stage							
I - III	5% (6)	5% (3)	5% (3)	1.00	0%	13% (3)	0.045
IV	95% (120)	95% (60)	95% (60)	1.00	100% (40)	87% (20)	0.045
Disease Extent (# organs involved)	2.7 ± 2.0	3.1 ± 2.1	2.4 ± 1.8	0.02	3.6 ± 2.0	2.3 ± 2.1	0.02
Anti-Cancer Regimen							
Chemotherapy							
Alkylating agent	32% (40)	29% (18)	36% (22)	0.48	31% (12)	26% (6)	0.70
Platinum	36% (45)	41% (26)	30% (19)	0.25	50% (20)	26% (6)	0.06
Antimetabolite	37% (47)	40% (25)	35% (22)	0.71	38% (15)	44% (10)	0.64
Anthracycline	25% (32)	25% (16)	25% (16)	1.00	25% (10)	26% (6)	0.92
Mitotic inhibitor	37% (47)	37% (23)	38% (24)	1.00	35% (14)	39% (9)	0.74
Biologic agents	32% (40)	32% (20)	32% (20)	1.00	30% (12)	35% (8)	0.70
Radiation Therapy	36% (45)	37% (23)	35% (22)	1.00	35% (14)	39% (9)	0.74
Antiplatelet Therapy^b^	24% (30)	19% (12)	29% (18)	0.31	15% (6)	26% (6)	0.33
Anticoagulation Therapy^c^	26% (33)	35% (22)	18% (11)	0.04	30% (12)	16% (10)	0.28
Coronary Artery Disease	11% (14)	8% (5)	14% (9)	0.42	5% (2)	13% (3)	0.35
Atherosclerosis Risk Factors							
Hypertension	35% (44)	32% (20)	38% (24)	0.56	35% (14)	26% (6)	0.46
Diabetes mellitus	10% (12)	5% (3)	14% (9)	0.15	8% (3)	0% (0)	0.29
Hypercholesterolemia	26% (33)	21% (13)	32% (20)	0.25	15% (6)	30% (7)	0.20
Tobacco use	46% (58)	46% (29)	46% (29)	1.00	38% (15)	61% (14)	0.07
Cardiac Morphology and Function							
Left Ventricle							
Ejection fraction (%)	61 ± 12	63 ± 9	59 ± 15	0.09	63 ± 9	62 ± 10	0.51
Ejection fraction <50%	15% (19)	12% (7)	20% (12)	0.27	11% (4)	13% (3)	1.00
Stroke volume (mL)	70 ± 24	70 ± 25	70 ± 22	0.98	67 ± 23	74 ± 29	0.31
End-diastolic volume (mL)	119 ± 45	113 ± 43	125 ± 47	0.18	107 ± 38	122 ± 50	0.19
End-systolic volume (mL)	49 ± 34	43 ± 23	55 ± 42	0.06	40 ± 20	48 ± 28	0.19
End-diastolic diameter (cm)	4.7 ± 0.7	4.6 ± 0.7	4.8 ± 0.8	0.08	4.5 ± 0.6	4.8 ± 0.7	0.06
Myocardial mass (gm)	118 ± 55	121 ± 69	115 ± 37	0.53	126 ± 79	112 ± 51	0.44
Right Ventricle							
Ejection fraction (%)	53 ± 8	53 ± 9	53 ± 8	0.92	53 ± 9	54 ± 8	0.47
Ejection fraction <50%	17% (22)	22% (13)	15% (9)	0.45	27% (10)	13% (3)	0.33
Stroke volume (ml)	69 ± 26	69 ± 26	71 ± 25	0.69	66 ± 23	74 ± 31	0.27
End-diastolic volume (mL)	134 ± 50	129 ± 47	139 ± 52	0.23	127 ± 43	135 ± 55	0.49
End-systolic volume (mL)	64 ± 33	61 ± 26	67 ± 38	0.24	61 ± 26	62 ± 27	0.88
Atria							
Left atrial area (cm^2^)	20 ± 7	20 ± 7	20 ± 6	0.97	19 ± 7	21 ± 8	0.40
Right atrial area (cm^2^)	19 ± 7	19 ± 6	19 ± 7	0.94	19 ± 6	19 ± 6	0.71

^a^Other cancer etiologies for C_NEO_: melanoma/skin (13% [n = 5]), endocrine (10% [n = 4]), head/neck (5% [n = 2]), and breast (3% [*n* = 1])

^b^Aspirin or thienopyridine

^c^Warfarin, non-vitaming K oral anticoagulant, or full dose low molecular weight heparin

Regarding comparisons between C_MASS_ subtypes, Table 1 demonstrates that C_NEO_ and C_THR_ differed with respect to cancer etiology: Among patients with C_THR_, lymphoma (30%) and gastrointestinal tumors (26%) were the most common underlying malignancies. Among patients with C_NEO_, sarcoma (20%) and lung (18%) were most common, although cancers not typically associated with cardiac involvement (e.g. endocrine, head and neck carcinomas) were also included in the study cohort. Whereas the majority of patients with C_NEO_ (100%) and C_THR_ (87%) had pre-existing stage IV cancer (irrespective of cardiac involvement), systemic disease burden –based on total number of non-cardiac organ systems involved - was higher among patients with C_NEO_ vs. those with C_THR_ (*p* = 0.02).

### Anatomic distribution and Sequelae

Table 2 compares anatomic distribution and sequelae of C_NEO_ and C_THR_. As shown, right-sided chamber involvement (i.e. RA or RV) occurred in the majority of patients with either condition, prevalence of which was similar between C_NEO_ and C_THR_ (*p* = 0.14). C_THR_ more commonly localized to the RA (78%; *p* < 0.001 vs. C_NEO_) – nearly all cases (17/18) of right atrial C_THR_ were associated with central venous catheters inserted for chemotherapy administration. Whereas nearly half (43%) of patients with C_NEO_ had RV involvement (*p* = 0.001 vs. C_THR_), individual chamber location was highly variable. Regarding distribution, rates of multi-chamber involvement tended to be higher among C_NEO_ affected patients (23% vs. 4%, *p* = 0.08).

**Table 2:** Anatomic Features and Sequelea

	C_NEO_	C_THR_	p
Anatomic Distribution			
Chamber Involvement			
Right atrium	25% (10)	78% (18)	<0.001
Right ventricle	43% (17)	4% (1)	0.001
Left atrium	15% (6)	4% (1)	0.41
Left ventricle	28% (11)	17% (4)	0.36
Right atrium or right ventricle	60% (24)	78% (18)	0.14
Multichamber involvement^a^	23% (9)	4% (1)	0.08
Pericardial involvement	30% (12)	0% (0)	0.002
Valvular adherence			
Outflow tract or valvular stenosis	13% (5)	0% (0)	0.15
Valvular regurgitation	20% (8)	17% (4)	1.00
Effusion			
Pericardial	25% (10)	17% (4)	0.48
Pleural	53% (21)	17% (4)	0.006

^a^Among C_NEO_ patients with multichamber involvement (23% [*n* = 9]), anatomic distribution was as follows: Left and right ventricle (8% [n = 3]); right atrium and right ventricle (8% [*n* = 3]); left atrium and left ventricle (3% [n = 1]); left and right atria (3% [*n* = 1]); left atrium, left ventricle and right ventricle (3% [n = 1])

Despite increased cardiac disease burden, assessed based on extent of chamber involvement and primary lesion size, C_NEO_ was rarely associated with functional impairment or localized effusions on CMR. For example, only 13% of C_NEO_ cases were associated with outflow tract or valvular stenosis, and only 25% were associated with pericardial effusions (8/10 in context of pericardial metastases).

### Tissue characterization

**Fig. 3:**
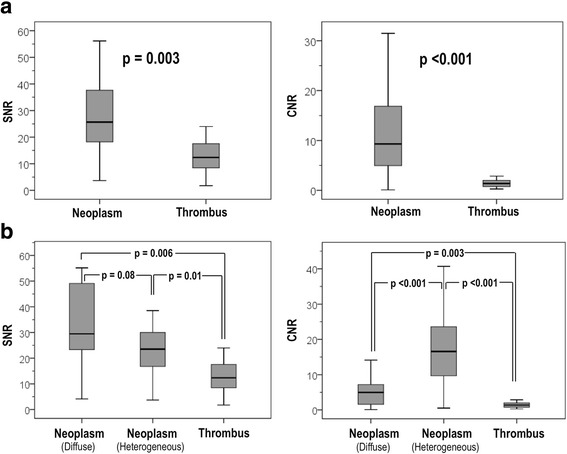
Quantitative Tissue Properties of Cardiac Neoplasm and Thrombus. **a** SNR (left) and CNR (right) compared between C_NEO_ and C_THR_ (data shown as overall distribution [line bars] together with 25–75% distribution [box], and median [central line]). Note that SNR and CNR were generally higher for C_NEO_, consistent with contrast-enhancement secondary to vascular supply. **b** SNR and CNR comparisons inclusive of C_NEO_ subtypes (diffuse and heterogeneous enhancement). Increased CNR within heterogeneously enhancing lesions (*p* < 0.001 vs. other types) consistent with interspersed regions with and without adequate vascular supply

Figure 3a compares SNR and CNR between visually scored C_NEO_ and C_THR_. As shown, both quantitative indices were higher within C_NEO_ vs. C_THR_ (SNR 29.7 ± 20.4 vs. 15.0 ± 11.4 | CNR 13.1 ± 13.0 vs. 1.6 ± 1.0; both *p* < 0.01), consistent with increased contrast uptake due vascular supply. Regarding C_NEO_ subtypes, data shown in Fig. 3b indicate that lesions with diffuse enhancement tended to have higher SNR than did those with heterogeneous enhancement, although this was not statistically significant (38.3±27.5 vs. 24.0±11.7; *p* = 0.08): Neoplasm with either enhancement pattern had higher SNR than did C_THR_ (15.0 ± 11.4; both *p* < 0.05). CNR was higher among lesions with visually scored heterogeneous enhancement (18.3±14.3) compared to either diffusely enhancing C_NEO_ (5.2±3.9; *p* < 0.001) or C_THR_ (1.6±1.0; p < 0.001), consistent with interspersed regions of tissue vascularity (enhancement) and tissue necrosis (non-enhancement).

**Fig. 4:**
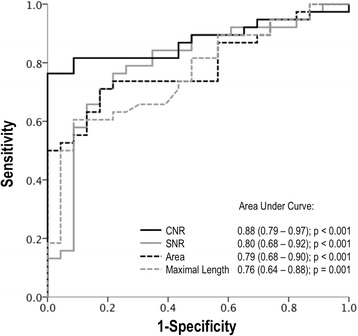
Receiver Operating Characteristics Curves. ROC curves for CNR, SNR, and lesion size (length, area) as indices for discriminating between C_MASS_ types. As shown, CNR yielded highest overall diagnostic performance (based on area under the curve [AUC]) for differentiating between C_NEO_ and C_THR_. AUC associated *p*-values reflect comparisons to null hypothesis (area = 0.5)

Tissue characterization differences between cardiac masses were paralleled by differences in anatomic features. As shown in Table 3, overall comparisons between C_NEO_ and C_THR_ demonstrated the former to typically be larger, whether assessed based on area or linear dimensions (both p < 0.05). However, further stratification demonstrated differences to vary based on C_NEO_ pattern of enhancement: Neoplastic lesions with heterogeneous enhancement tended to be larger than those with diffuse enhancement, whether quantified by area (*p* < 0.001) or linear dimensions (*p* < 0.1). Of note, while all anatomic indices were larger for heterogeneously enhancing lesions compared to C_THR_, (*p* < 0.005), diffusely enhancing C_NEO_ lesions and C_THR_ were not significantly different in size (*p* > 0.05). Figure 4 illustrates ROC curves concerning overall performance of CNR, SNR, and lesion size (area, maximal length) for differentiation between C_MASS_ subtypes (C_NEO_, C_THR_). Table 4 reports diagnostic test variables calculated using cutoffs derived from corresponding ROC curves. As shown, AUC (0.88 [0.79–0.97]) and diagnostic accuracy (85%) were highest for CNR, consistent with use of contrast-enhancement as the criterion for C_NEO_.

**Table 3:** Tissue Characteristics in Relation to Anatomic Properties

	C_NEO_	C_THR_	*p*	C_NEO_		*p* (HETERO VS DIFFUSE)	*p* (HETERO VS. THR)	*p* (DIFFUSE VS. THR)
				C_NEO-HETERO_ (*n* = 25)	C_NEO-DIFFUSE_ (*n* = 15)			
Area (cm^2^)	17.3 ± 23.8	2.0 ± 1.5	<0.001	25.8 ± 26.6	3.0 ± 2.7	<0.001	<0.001	0.21
Perimeter (cm)	16.0 ± 13.3	5.9 ± 2.7	<0.001	21.6 ± 13.9	6.6 ± 2.6	<0.001	<0.001	0.45
Maximal Length (cm)	5.8 ± 4.9	2.3 ± 1.6	<0.001	7.0 ± 3.8	3.9 ± 5.9	0.06	<0.001	0.22
Orthogonal Length (cm)	3.3 ± 2.5	2.0 ± 2.0	0.04	4.1 ± 2.5	2.0 ± 2.1	0.01	0.003	0.96
Perimeter/Min Length	5.3 ± 2.1	4.6 ± 2.8	0.26	5.7 ± 2.3	4.7 ± 1.4	0.13	0.15	0.94

**Table 4:** Diagnostic Test Performance in Relation to Quantitative Signal Intensity and Lesion Size^a^

	Sensitivity	Specificity	Accuracy	Positive Predictive Value	Negative Predictive Value
Signal Intensity Variables					
Contrast-to-noise ratio	76% (29/38)	100% (23/23)	85% (52/61)	100% (29/29)	72% (23/32)
Signal-to-noise ratio	71% (27/38)	83% (19/23)	75% (46/61)	87% (27/31)	63% (19/30)
Lesion Size Variables					
Area (cm^2^)	73% (29/40)	83% (19/23)	76% (48/63)	88% (29/33)	63% (19/30)
Maximal length (cm)	63% (25/40)	91% (21/23)	73% (46/63)	93% (25/27)	58% (21/36)

^a^Cutoffs derived (for maximum sensitivity and specificity) from ROC curves shown in Fig. 4 (parameter-based cutoffs as follows: CNR 4.50, SNR 19.36, area 2.76, maximum length 3.27)

### Clinical outcomes

Among patients with C_NEO_, 8% (*n* = 3) underwent resection, 43% (*n* = 17) had a change in chemotherapy regimen and 13% (*n* = 5) underwent targeted radiation of the heart and/or mediastinum within 6 months after CMR. Less than half of C_NEO_ patients were treated with anticoagulation, as compared to nearly all patients with C_THR_ (38% vs. 96%, *p* < 0.001). Regarding embolic events, pulmonary embolism was more common among patients with C_MASS_ (18% vs. 6%, *p* = 0.12), as well as among patients with right sided C_MASS_ + compared to controls (C_MASS_ -) or C_MASS_ + patients with isolated left sided involvement (24% vs. 6%, *p* = 0.004): Among C_MASS_ sub-types, pulmonary embolism rates were similarly high among patients with C_NEO_ (20%) and C_THR_ (13%). Rates of cerebrovascular accident were identical between patients with and without C_MASS_ (6% vs. 5%, *p* = 1.00), and did not differ when patients were further stratified by left sided C_MASS_ location (10% vs. 5%, *p* = 0.31).

**Fig. 5:**
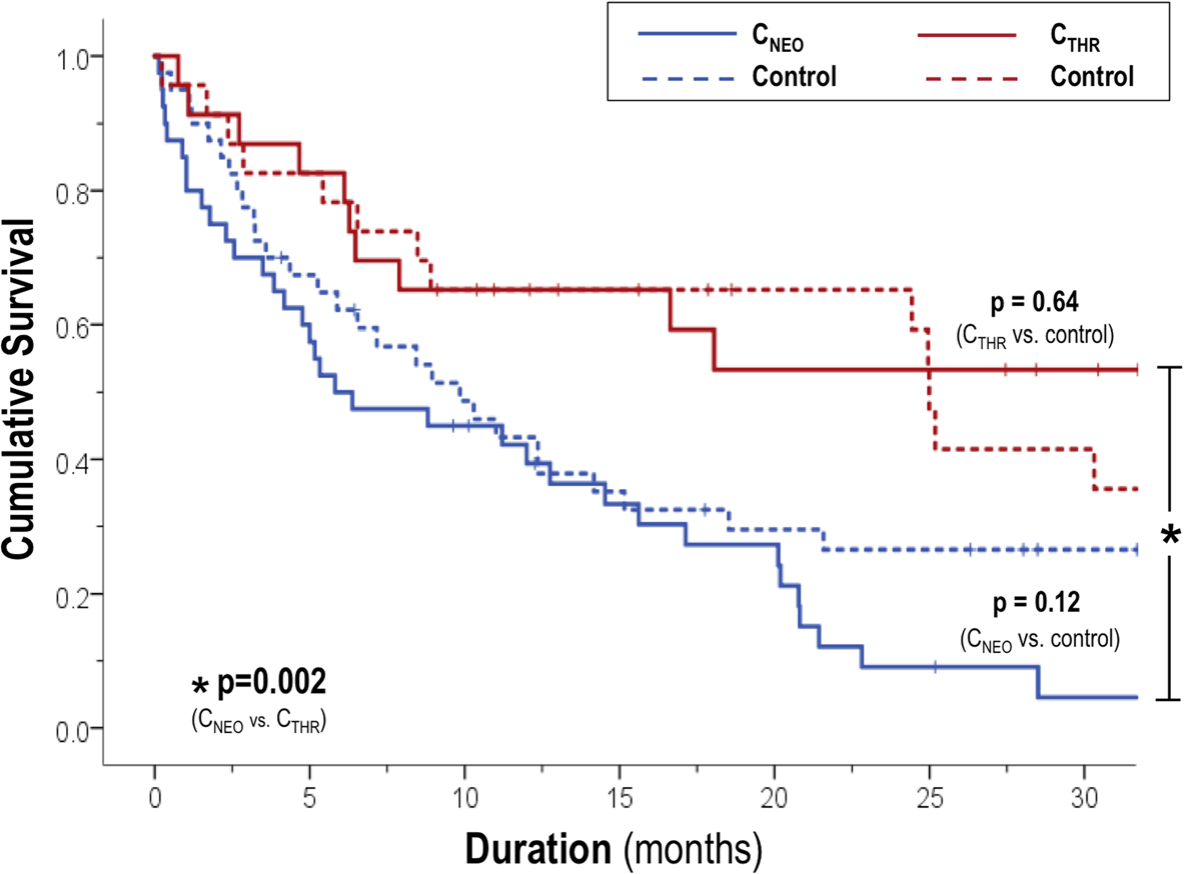
Mortality Status. Kaplan Meier survival curves for patient groups partitioned based on C_MASS_ status (solid blue = C_NEO_, dotted blue = C_NEO_ control; solid red = C_THR_, dotted line = C_THR_ control): For both C_NEO_ and C_THR_, controls were matched for primary cancer type and stage. Note higher mortality among patients with C_NEO_ vs. C_THR_ (*p* = 0.002); C_THR_ conferred similar mortality risk compared to respective cancer-matched controls whereas mortality associated with C_NEO_ was slightly higher albeit non-significant

Patient mortality was assessed following CMR to test the impact of C_MASS_ related tissue properties on clinical prognosis. Median duration of post-CMR follow-up was 2.5 years (IQR 1.1–3.8) among survivors; median survival after imaging was 1 year. Figure 5 provides Kaplan Meier survival curves of C_NEO_ and C_THR_ affected patients, as well as controls (C_MASS_) matched for primary cancer type and stage. As shown, mortality risk was similar between C_THR_ affected patients and controls (hazard ratio [HR] = 0.82 [CI 0.35–1.89], *p* = 0.64). In contrast, C_NEO_ affected patients tended towards slightly higher mortality compared to controls, although differences were non-significant (HR = 1.50 [CI 0.90–2.49], *p* = 0.12). Risk for death by 6 months post-CMR among C_NEO_ and C_THR_ patients compared to cancer-matched controls without cardiac involvement were (C_NEO_: 50% vs. 38% | C_THR_: 22% vs. 22%); corresponding risks at 1 year were proportionately higher (C_NEO_: 61% vs. 57% | C_THR_: 35% vs. 35%).

Comparisons between C_NEO_ and C_THR_ affected patients demonstrated prognosis to be markedly worse among the former (HR = 3.13 [CI 1.54–6.39], *p* = 0.002); mortality was approximately 2-fold higher among patients with C_NEO_ at 6 months (50% vs. 22%) and at 1-year (61% vs. 35%) post-CMR. Of note, C_NEO_ was associated with increased mortality risk, whereas lesion size – as assessed via area (HR = 0.99 per cm^2^ [CI 0.98–1.01], *p* = 0.40) or maximal diameter (HR = 0.98 per cm [CI 0.91–1.06], *p* = 0.61) was not. Outcomes were not significantly different between C_NEO_ patients with heterogeneous and diffusely enhancing lesions (HR = 1.14 [CI: 0.60–2.26], *p* = 0.70). Similarly, among the small number of patients with multichamber involvement mortality did not statistically differ compared to C_MASS_ + patients with lesions confined to a single cardiac chamber (HR = 1.40 [CI 0.62–3.16], *p* = 0.41).

## Discussion

This is the largest study to date examining anatomic pattern, tissue properties, and differential prognostic implications of CMR-evidenced cardiac masses (C_MASS_ +) among patients with systemic cancer. Key findings are as follows. First, among a broad cancer cohort for which C_MASS_ + etiology was defined based on presence or absence of contrast enhancement on LGE-CMR, likelihood of C_NEO_ paralleled extra-cardiac disease burden – as evidenced by higher total number of non-cardiac organ systems involved among patients with C_NEO_ vs. C_THR_ (*p* = 0.02). Second, whereas C_THR_ was classified based on uniform absence of enhancement, two distinct C_NEO_ patterns were identified - heterogeneous and diffuse enhancement. CNR was highest among lesions with heterogeneous enhancement (*p* < 0.001) - consistent with interspersed regions of tissue vascularity and tissue necrosis. C_NEO_ lesions with heterogeneous enhancement were larger than C_NEO_ lesions with diffuse enhancement, as well as C_THR_ (both *p* < 0.05). Conversely, diffusely enhancing C_NEO_ lesions and C_THR_ were of similar size (*p* = NS). Finally, follow-up data demonstrated C_THR_ to confer similar mortality risk compared to cancer-matched controls without cardiac involvement (HR = 0.82 [CI 0.35–1.89], *p* = 0.64) whereas mortality among C_NEO_ affected patients was slightly higher but not significantly different vs. matched controls (HR = 1.50 [CI 0.90–2.49], *p* = 0.12). Follow-up data also showed mortality to be increased among patients with LGE-CMR defined C_NEO_ compared to those with C_THR_ (HR = 3.13 [CI 1.54–6.39], *p* = 0.002); outcomes were similar when patients were stratified based on lesion size (HR = 0.99 per cm^2^ [CI 0.98–1.01], *p* = 0.40).

Regarding the diagnostic approach employed in our study, it is important to recognize the concept that C_NEO_ can be distinguished from C_THR_ based on contrast-enhancement is not modality specific: For example, Kirkpatrick et al. - studying a cohort in whom pathology and anticoagulation response were respectively used to verify C_NEO_ and C_THR_, reported that contrast uptake on perfusion echocardiography was uniformly associated with malignant C_NEO_ whereas hypo-enhancement was associated with C_THR_ [13]. Given the established concept that C_NEO_ manifests contrast-enhancement due to intrinsic vascular supply, and that vascularity is a key component for cellular proliferation/lesion growth, our finding that C_NEO_ were generally larger than C_THR_ is consistent with general concepts in tumor biology, for which lesion growth has been shown to correlate with vascular supply [14–16]. Our results also show that cancer-associated enhancement on LGE-CMR can vary in pattern, manifesting as diffuse or heterogeneous enhancement. The notion that heterogeneous enhancement on CMR is a marker of tissue necrosis has also been demonstrated via non-cardiac magnetic resonance imaging (MRI): Among patients with hepatic cell carcinoma, central hypo-enhancement on liver MRI has been shown to correspond to pathology-evidenced coagulation necrosis [17]. Regarding mechanism, in-vitro and ex-vivo studies have shown tumor necrosis to stem from mismatch between tumor growth and vascular supply, leading to cell death and tissue necrosis [18, 19]. It is possible that heterogeneous enhancing C_NEO_ may be partially attributable to surface thrombosis, as can be superimposed on necrotic and/or hypercoagulable tissue. Whereas our study did not directly perform serial imaging to directly assess tumor growth or therapeutic response, our finding of increased lesion size among patients with heterogeneous compared to diffusely enhancing C_NEO_ is consistent with the notion that heterogeneous enhancement stems from underlying differences in tumor growth.

Our current findings add to growing literature demonstrating C_MASS_ + tissue characterization to provide diagnostic and prognostic utility among cancer and non-cancer cohorts. Prior data from our group has shown an association between LV thrombus (defined by LGE-CMR) and risk for embolic events among heart failure cohorts [6, 7]. Similarly, multicenter clinical trial data has shown LGE-CMR evidenced LV thrombus to predict all cause mortality [20]. Among patients with advanced cancer, recent data from our group has shown C_NEO_ to be associated with poor prognosis (44% 6-month mortality) [3]. However, this analysis was limited to patients with LGE-CMR defined C_NEO_, thereby precluding study of differential prognosis associated with presence or absence of lesion-associated contrast-enhancement. Our current study addresses this key knowledge gap – findings support incremental utility of tissue characterization via LGE-CMR (vs. anatomic assessment via techniques such as cine-CMR or echo) to guide therapeutic decision-making and prognostic risk stratification for cancer-patients with cardiac masses.

It is noteworthy that while mortality rates markedly differed between patients with C_NEO_ and C_THR_, prognosis of each group was similar to that of cancer-affected controls (C_MASS_ -) matched for disease etiology and extent of extra-cardiac disease. Regarding C_THR_, we speculate that this is attributable to the fact that this condition is treatable (via anticoagulation) and that the majority of thrombosis was limited to the right atrium and thus not exposed to high pressure, systemic circulatory conditions predisposing to life-threatening embolization. Consistent with this notion, our findings suggest that patients with C_THR_ on LGE-CMR were near uniformly treated with anticoagulants (96%). Regarding C_NEO_, our finding of a numerically higher although non-significant mortality rates vs. controls (*p* = 0.12) suggests that the primary determinant of outcome relates to cancer etiology and burden of systemic disease, for which cardiac involvement is only one component similar to that of other organ systems.

Several limitations should be noted. First, our study population was derived from patients with C_MASS_ referred for clinical CMR at a single tertiary care cancer center: C_MASS_ affected cases and controls were specifically matched for cancer etiology and extent of extra-cardiac disease to test the additive impact of presence and type of C_MASS_ on survival. In this context, it is important to recognize that mortality rates among controls may not reflect those of a general population of advanced cancer patients, but rather survival in a select group for which cancer etiology and stage were similar to that of affected (C_MASS_ +) cases. Mortality estimates should also be interpreted keeping in mind that our study included patients at various times after their diagnoses and only evaluated patients who were healthy enough to undergo CMR. Second, this study used LGE-CMR to define C_MASS_ type (i.e. neoplasm or thrombus) based on presence or absence of contrast uptake so as to test an established imaging approach well validated based on prior research by our group and others [3, 6–9, 20]. Alternative imaging strategies such as perfusion and T1 mapping can also measure contrast-enhancement in a manner similar to LGE-CMR – these methods were not tested in our study, but hold potential for quantitative assessment of C_MASS_ associated enhancement. Third, our estimates of diagnostic test performance (e.g. accuracy) for given imaging parameters (e.g. CNR, SNR) were assessed using cutoff values chosen from the same data and are likely optimistic. Finally, it should be noted that our study included a broad array of patients with different primary cancer diagnoses. Whereas C_MASS_ + patients were matched (1:1) to C_MASS_ - patients with equivalent cancer type and stage so as to test impact of presence and type of C_MASS_ on prognosis, heterogeneity in cancer etiology is a potential confounding variable that could have impacted our results. Further larger studies in uniform cancer populations are needed to examine impact of C_MASS_ tissue properties on cancer-associated outcomes.

## Conclusions

Findings of this study demonstrate that among cancer patients with C_MASS_, presence or absence of LGE-CMR evidenced contrast-enhancement is a powerful prognostic indicator: C_NEO_ as defined by LGE-CMR tissue characterization conferred markedly poorer prognosis than C_THR_, whereas anatomic assessment of lesion size via cine-CMR did not stratify mortality risk. Both C_NEO_ and C_THR_ are associated with similar prognosis compared to C_MASS_ - controls matched for cancer type and disease extent. Future, multicenter research among patients with C_NEO_ is warranted to test whether prognosis or therapeutic response varies based on pattern or extent of enhancement as measured by LGE-CMR or emerging CMR tissue characterization approaches.

## Abbreviations

AUC: Area under the curve
bSSFP: Balanced steady-state free precession
CI: Confidence interval
C_MASS_: Cardiac mass
CMR: Cardiovascular magnetic resonance
C_NEO_: Cardiac neoplasm
CNR: Contrast-to-noise ratio
C_THR_: Cardiac thrombus
HR: Hazard ratio
IQR: Interquartile range
IR-GRE: Inversion recovery gradient recalled echo
LA: Left atrium/left atrial
LGE-CMR: Late gadolinium enhancement cardiovascular magnetic resonance
LV: Left ventricle/left ventricular
MRI: Magnetic resonance imaging
RA: Right atrium/right atrial
ROC: Receiver Operating Characteristics Curves
RV: Right ventricle/right ventricular
SNR: Signal-to-noise ratio
TI: Inversion time
